# Hetero-deformation induced stress and its microstructural morphology dependence in a near-alpha titanium alloy

**DOI:** 10.1371/journal.pone.0356747

**Published:** 2026-08-24

**Authors:** Lin Xie, Jialian Li, Yuan Xing, Yuhui Wang

**Affiliations:** 1 School of Advanced Materials, Chengdu Aeronautic Polytechnic University, Chengdu, Sichuan, China; 2 Key Laboratory of the Evaluation and Monitoring of Southwest Land Resources (Ministry of Education), Sichuan Normal University, Chengdu, Sichuan, China; 3 State Key Laboratory of Vanadium and Titanium Resources Comprehensive Utilization, Pangang Group Research Institute Co., Ltd., Chengdu, Sichuan, China; 4 National Engineering Research Center for Equipment and Technology of Cold Strip Rolling, Yanshan University, Qinhuangdao, Hebei, China; Malla Reddy (MR) Deemed to be University, INDIA

## Abstract

Hetero-deformation induced (HDI) stress and the effect of microstructural morphology on it were investigated in a near-alpha titanium alloy featuring lamellar and equiaxed initial microstructures. The development of HDI stresses were confirmed in the two types of microstructures under tensile tests. While both microstructures exhibit comparable HDI stress magnitudes during initial tensile deformation, the equiaxed one demonstrates obviously enhanced HDI strain hardening compared to its lamellar counterpart as deformation progressed. Two factors are mainly responsible for the observed divergence in HDI stress evolution between the two microstructures. First and foremost is the surface-to-volume ratio of phase interface (*s*_V_), where the equiaxed structure shows significantly higher *s*_V_ than its lamellar counterpart, which can offer more potential sites and better spatial interconnectivity of phase interfaces for GNDs development. The other factor is the microstructural size scale, in which the average layer thickness of the lamellar structure is considered being too small, as compared to the average grain diameter in the equiaxed one, to provide sufficient space for GND pileups to fully develop, which limits HDI strain hardening. This finding provides critical insights into microstructural design strategies for optimizing the development of HDI stress.

## 1. Introduction

Microstructure is well recognized as a key factor in tailoring the mechanical properties of metals. Recently, heterogeneous microstructural design has attracted extensive scientific interest because of its potential in managing both strength and ductility. Microstructures of this type are characterized by heterogeneous regions with obviously different mechanical properties, including dual/multi-phase structure [1,2], bi/multi-modal structure [3,4], gradient structure [5], harmonic structure [6] and heterogeneous lamella structure [7], etc. This heterogeneity in mechanical properties from one microstructural region to another can be induced by the difference in crystal structure, chemical composition or grain size [8]. The superior mechanical properties of heterostructures over their homogeneous counterparts mainly originates from the development of hetero-deformation induced (HDI) stress, which is a result of the interaction and coupling between the hetero-regions [9].

Dislocation slip is the dominant mechanism of plastic deformation in metals. It usually initiates in grains with slip planes having the highest Schmid factors and then transfers or be blocked by grain boundaries, depending on the orientation relationship between the neighboring grains [10]. The blocked slips can cause a high density of dislocations piled up against grain boundaries, resulting in the generation of geometrically necessary dislocations (GNDs) to accommodate the strain incompatibility between the adjacent grains [11]. In the case of heterostructure, however, such strain incompatibility is more pronounced due to the structural heterogeneity arising from the differences in mechanical properties between the hetero-regions. The interfaces dividing the microstructure into hetero-regions are different from grain boundaries in homogeneous microstructures. Large accumulation of GNDs against the hetero-interface can generate localized internal stress that eventually develops into a back stress [12] in the softer region. The back stress always runs in the reverse direction of the applied stress and thus can decrease the effectively resolved shear stress for the slip of dislocation [13]. Under the back stress, a forward stress will be induced in the harder region, which is in the same direction as the applied stress [14]. Both back stress and forward stress interacting at the hetero-interface with opposite directions will result in an HDI stress [14]. This long-range internal stress, where early literature referred to it as back stress alone, is mainly responsible for the strengthening and strain hardening mechanisms in heterostructured metals [15].

Since HDI stress is produced by GND pileups at hetero-interfaces, it naturally speculates that the change in the arrangement of such interfaces, which is a result of microstructural morphology variation, could lead to a change in HDI stress. However, recent studies relating to the hetero-interface were mainly focused on the strain gradient [16], interface-affected zone (IAZ) [17] and size effect [18] on the hetero-deformation behavior. Few attentions have been paid to the influence of microstructural morphology on the development of HDI stress, which is fundamental in determining the mechanical properties of heterostructured metals.

In this work, a near-alpha titanium alloy, consisting of a hexagonal-close-packed α matrix and a body-centered cubic β phase, was chosen as a model material to study the effect of microstructural morphology on HDI stress. Such an alloy exhibits excellent microstructural tunability, with well-developed thermomechanical processes to produce microstructures with fully lamellar or equiaxed morphologies, which represent the two fundamental and contrasting morphological types for most metals. Critically, these processing routes allow for a direct comparison of morphology effects without significantly altering the overall chemical composition or the volume fraction of the constituent phases. Moreover, the near-α titanium alloy undergoes virtually no β → α’ martensitic transformation at room temperature, thereby eliminating the potential influence of phase transformation and enabling a pure investigation of the role of microstructural morphology in governing the development of HDI stress.

## 2. Materials and methods

A hot-forged near-α titanium alloy was used as the starting material to produce two types of initial microstructures, with chemical composition of 6.60% Al, 1.59% Mo, 2.30% V, 2.24% Zr, 0.01% C, 0.03% Fe, 0.01% Si, 0.10% O, 0.04% N, and balanced Ti (mass%). Two samples were cut from the hot-forged bar. One was annealed at 1020°C for 50 min followed by furnace cooling to form a lamellar structure, and the other was annealed at 930°C for 60 min followed by furnace cooling to form an equiaxed structure.

The initial microstructures and the deformed microstructures after tensile tests were characterized by electron backscatter diffraction (EBSD) and transmission electron microscopy (TEM), respectively. EBSD mappings were performed using a JSM 7100F scanning electron microscope operated at an accelerating voltage of 20 kV and a step size of 200 nm. Samples for EBSD characterization were mechanically and electrochemically polished. TEM observations were conducted using a JEM 2100F TEM operated at an accelerating voltage of 200 kV. Specimens for TEM characterization were prepared by mechanical polishing to a thickness of 50 μm, followed by electropolishing using a twin-jet polisher.

Mechanical behaviors of alloys with different initial microstructures were characterized by uniaxial tensile tests using an Instron 5982 testing system. Tensile specimens of gauge dimensions 25 × 5 mm^2^ were tested with a constant strain rate of 6.67 × 10^−4^s^−1^ at room temperature. Tensile unloading-reloading tests were conducted to investigate the Bauschinger effect [19] and calculate the HDI stress, where the specimens were unloaded at a pre-designed strain by the stress-control mode to 20 N at the unloading rate of 200 N·min^-1^, and then reloaded to the same applied stress before the next cycle. Mechanical properties of the constituent phases were characterized by hardness values measured during interrupted tensile tests at different strain levels. The measurements were performed in the central region of the tensile specimen’s gauge section using a Keysight Nano Indenter G200 with a maximum load of 5 mN.

## 3. Results and discussion

**Fig 1:**
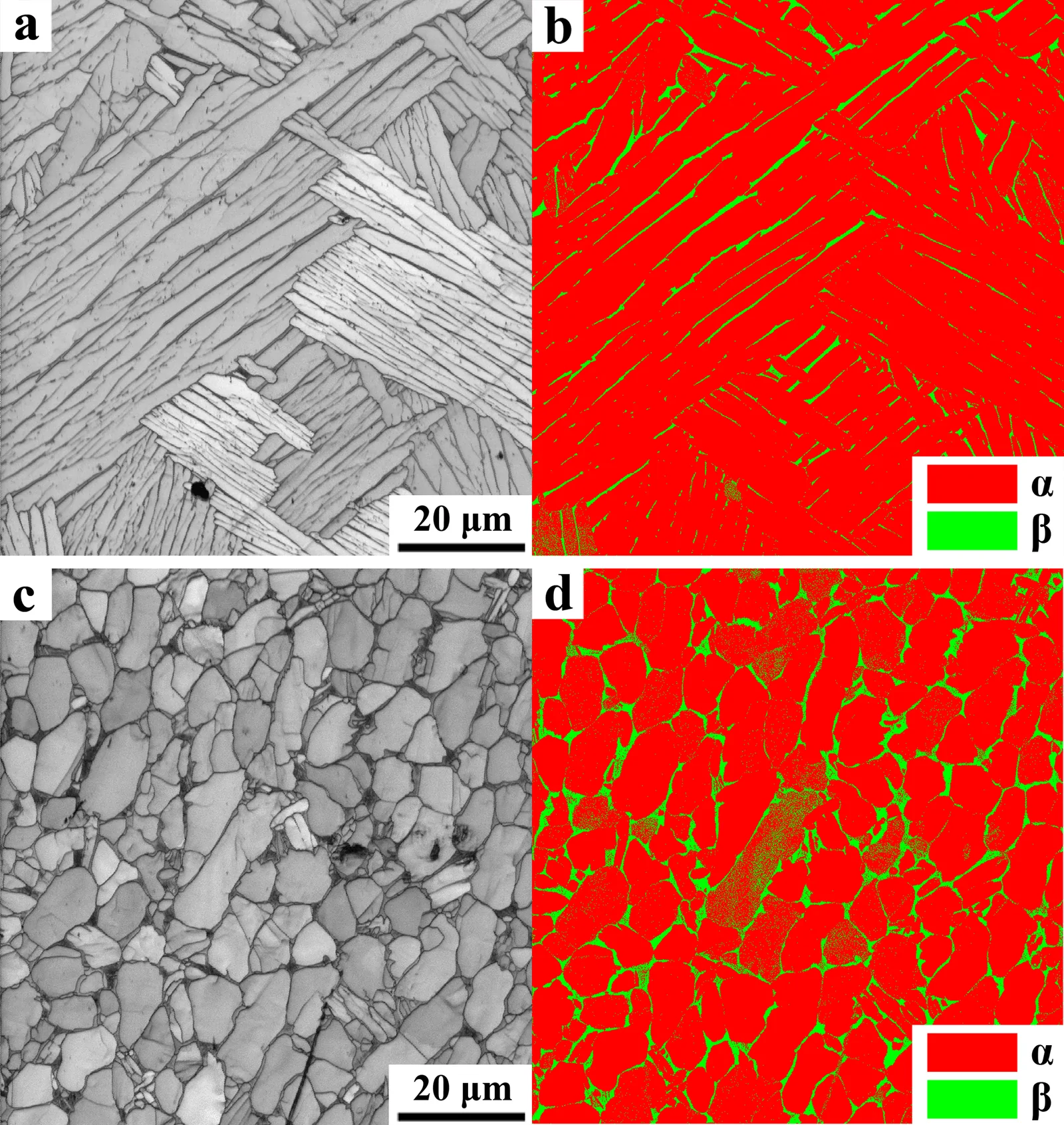
EBSD images of the as-received alloys. (a) band contrast map and corresponding (b) phase map of the lamellar structure; (c) band contrast map and corresponding (d) phase map of the equiaxed structure.

Fig 1 shows the EBSD maps for the two types of initial microstructures. Both of them are constituted by major α phase and minor β phase. The band contrast map shown in Fig 1a and the corresponding phase map shown in Fig 1b exhibit a typical lamellar morphology consisting of alternating thick α bands and thin β layers. The volume fraction (*f*) of the β phase is 8.3%. The average thickness (*t*) is 2.8 μm for the α bands and 0.9 μm for the β layers. On the other hand, the band contrast map and the corresponding phase map shown in Fig 1c and 1d clearly reveal an equiaxed morphology, in which the β phase is located at the grain boundaries and triple junctions of the equiaxed α grains. The volume fraction of the β phase is slightly higher than that of the lamellar structure, which was measured to be 11.8%. For the α grains, the average diameter (*d*) is 7.9 μm, and for the β films, the average thickness is 1.1 μm. Table 1 summarizes the microstructural parameters mentioned above. Also included in this table are the chemical compositions of each phase obtained by energy-dispersive spectrometer. It is seen that all alloying elements except Al show lower contents in the α phase than the β phase. There are no significant differences in the chemical compositions between the two types of microstructures.

**Table 1:** Microstructural parameters and chemical compositions of the as-received alloys.

Sample	Phase	Microstructural parameter			Chemical compostion (wt.%)				
		*f* (%)	*t* (μm)	*d* (μm)	Al	Mo	V	Zr	Ti
**Lamellar**	**α**	91.7	2.8 ± 1.2	———	6.82 ± 0.24	0.28 ± 0.06	1.07 ± 0.09	2.19 ± 0.26	Bal.
	**β**	8.3 ± 0.5	0.9 ± 0.3	———	3.36 ± 0.27	1.61 ± 0.22	2.68 ± 0.13	3.53 ± 0.28	Bal.
**Equiaxed**	**α**	88.2	———	7.9 ± 2.6	6.97 ± 0.51	0.33 ± 0.14	1.14 ± 0.11	2.08 ± 0.32	Bal.
	**β**	11.8 ± 1.4	1.1 ± 0.4	———	3.04 ± 0.18	1.74 ± 0.19	2.92 ± 0.34	3.98 ± 0.39	Bal.

**Fig 2:**
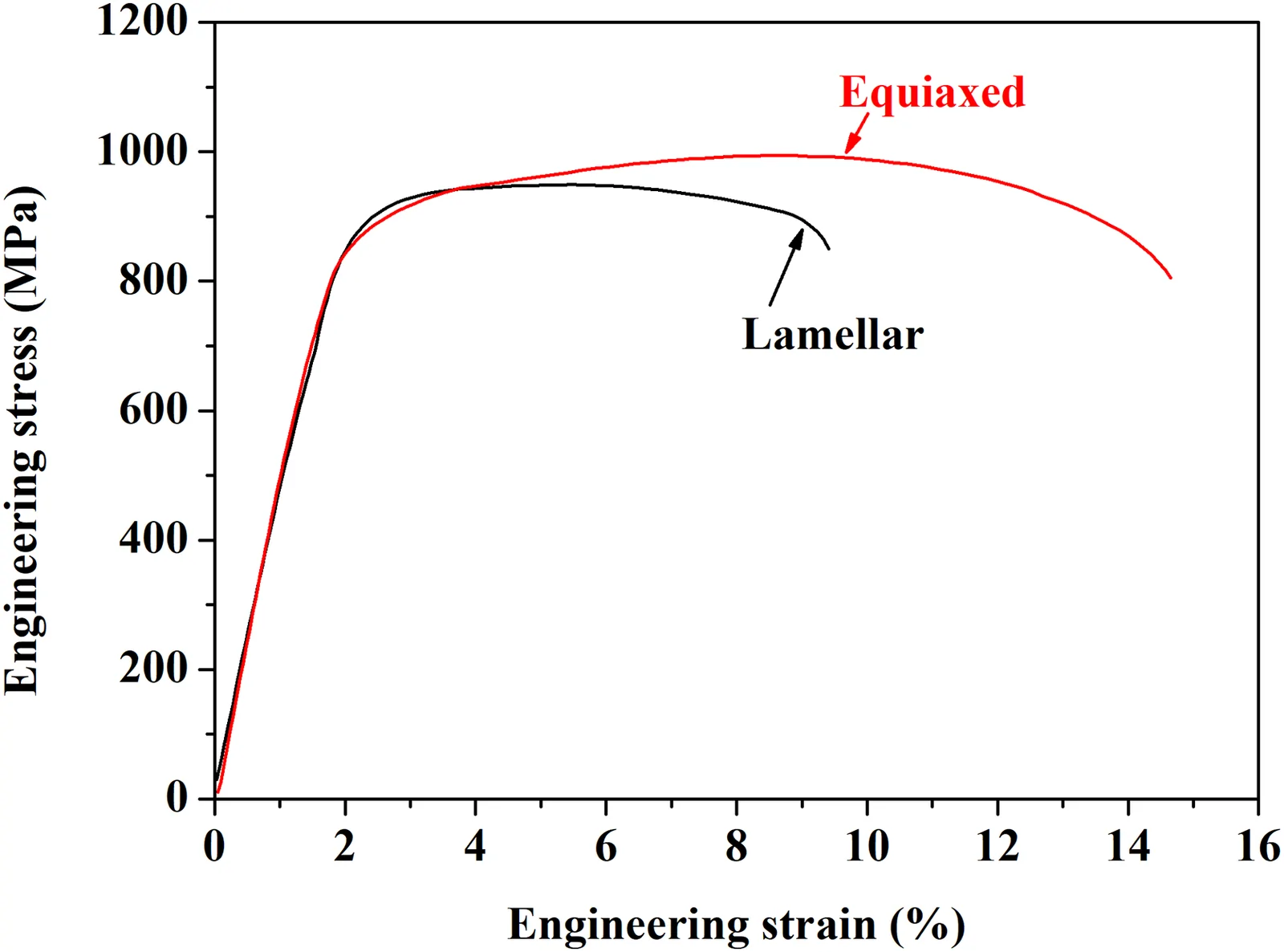
Engineering stress-strain curves of the alloys with lamellar and equiaxed structures.

Fig 2 shows the engineering stress-strain curves of the alloys with lamellar and equiaxed structures. Both curves exhibit continuous yielding without an obvious yield plateau. The tensile properties for both samples are summarized in Table 2. It seems that the morphological difference of initial microstructures has a limited effect on strength but a significant influence on ductility. The equiaxed structure shows a yield strength (*σ*_0.2_) of 842 MPa and a wide strain hardening stage that leads to a uniform elongation (*ɛ*_u_) of 9.1% and a total elongation (*ɛ*_t_) of 14.7%. On the other hand, the lamellar structure exhibits a slightly higher yield strength of 873 MPa, which can be attributed to its smaller layer thickness as compared to the grain diameter of the equiaxed structure. Note that the strain hardening stage of the lamellar structure is obviously narrower than that of the equiaxed one. The poorer strain hardening ability of the lamellar structure not only causes a lower ultimate tensile strength (*σ*_b_), 940 MPa as compared to 994 MPa of its equiaxed counterpart, but it also leads to an earlier tensile instability which occurs at a strain of 5.1% and finally, results in a limited total elongation of 9.3%.

**Table 2:** Tensile properties of the alloys with lamellar and equiaxed structures.

Sample	*σ*_0.2_ (MPa)	*σ*_b_ (MPa)	*ɛ*_u_ (%)	*ɛ*_t_ (%)
**Lamellar**	873 ± 7	940 ± 6	5.1 ± 0.2	9.3 ± 0.2
**Equiaxed**	842 ± 4	994 ± 8	9.1 ± 0.1	14.7 ± 0.2

**Fig 3:**
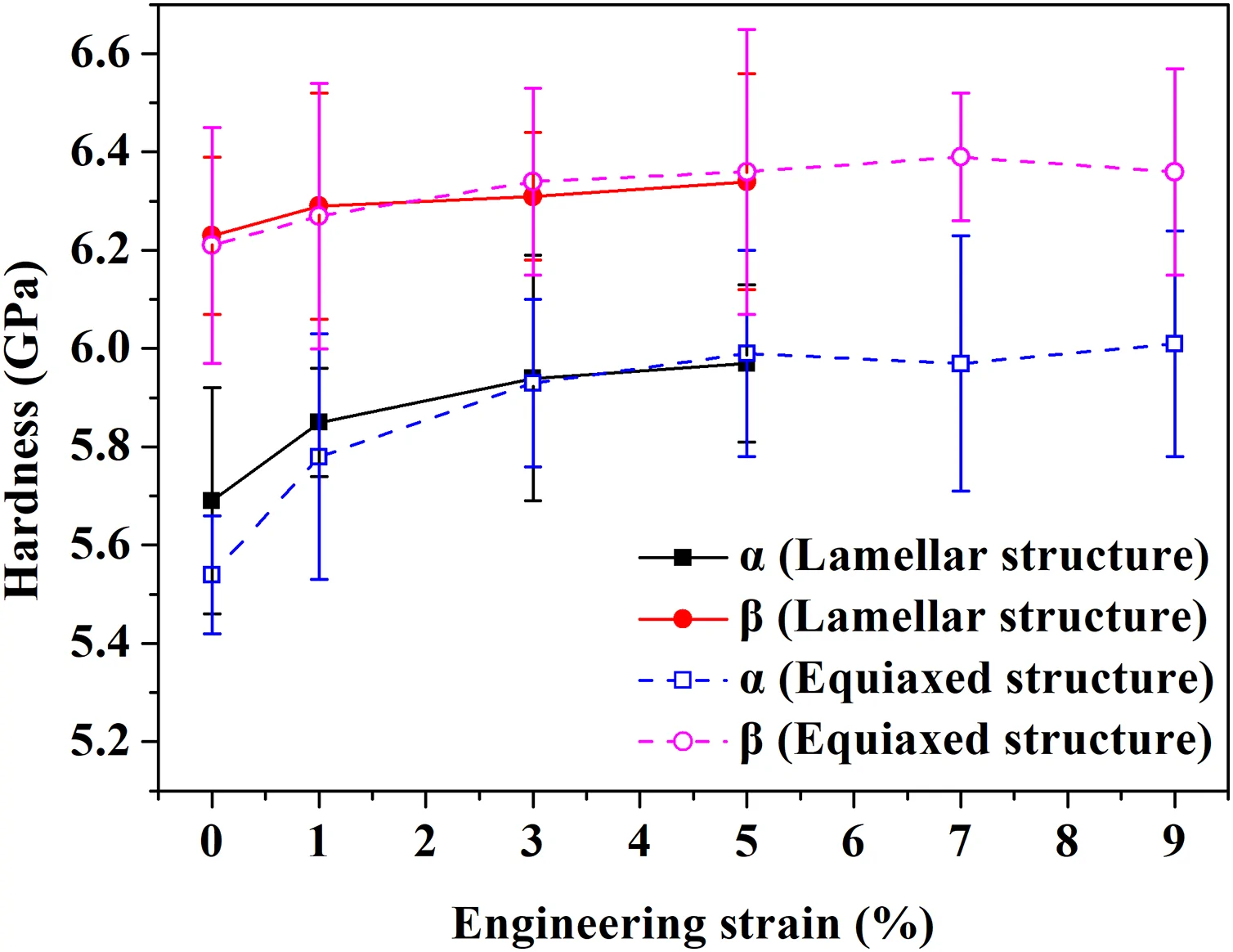
Hardness vs. engineering strain of the constituent phases in the alloys with lamellar and equiaxed structures.

**Fig 4:**
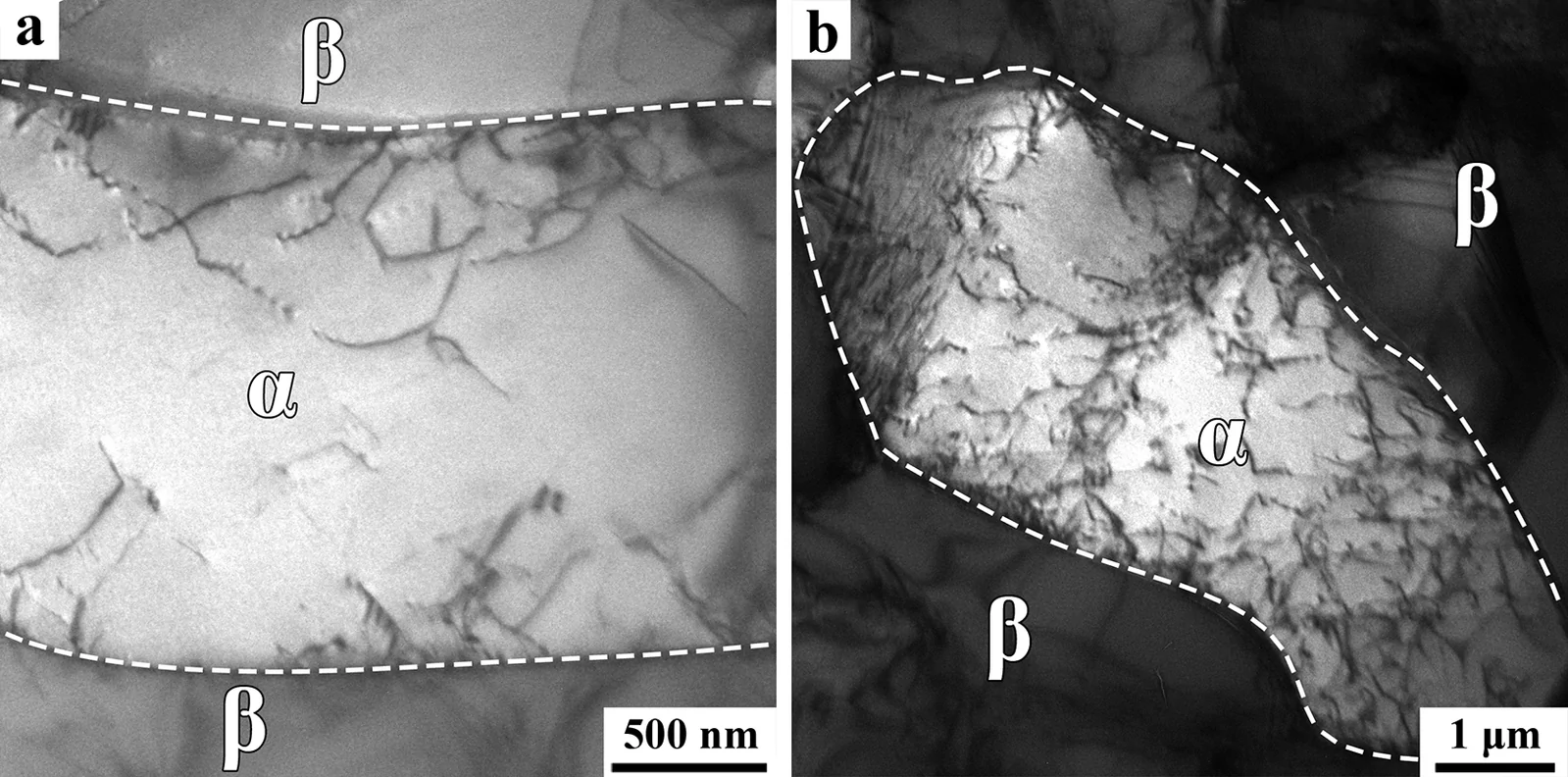
TEM bright-field images of the alloys after 1% tensile deformation, with the electron beam approximately parallel to the [11$\overset{―}{2}$0] zone axis. (a) lamellar structure; (b) equiaxed structure.

The above results suggest that the morphology of initial microstructure has a clear influence on the mechanical behavior of the alloy studied, which may have its cause in the composite characteristic of plastic deformation [20]. Fig 3 shows the hardness variations of the constituent phases for the lamellar- and equiaxed-structured samples during tensile deformation. It is seen that in both samples, the hardness values of the two phases increase concurrently with the tensile strain and then reach saturation at a later stage of tensile deformation. A clear difference in the average hardness between the constituent phases can be observed, with the β phases always exhibiting higher average hardness than their α counterparts. On the other hand, the strain hardening rates of the β phases seem to be lower than those of the α phases, especially in the early stage of tensile deformation. For the β phase, the evolutions of average hardness in both samples are almost the same. In the case of the α phase, although the lamellar structure shows a higher initial average hardness than its equiaxed counterpart, the strain hardening rate in the former is obviously lower than that in the latter, and their average hardness eventually reaches almost the same level toward the end of tensile deformation. Fig 4 shows the TEM images of the two types of samples after 1% tensile deformation. In this early stage of deformation, the applied strain on micro-scales is primarily relaxed by the activities of dislocations [21]. For the lamellar-structured sample, as typically shown in Fig 4a, dislocations mainly existed in the α lamella, especially near the phase interfaces. Similar situation can also be observed in the equiaxed one, as representatively shown in Fig 4b, where dislocations are statistically much richer in the α grain as compared to its surrounding β grains. The hardness results combined with TEM characterization clearly suggest a composite deformation mode, that is, the constituent phases due to their different mechanical properties lead to a strain incompatibility between them and then results in a heterogeneous dislocation configuration. Quantification of the degree of the heterogeneous plastic deformation is therefore crucial to reveal how microstructural morphology affects the mechanical behavior.

In the present alloy, the α phase is softer than the β phase, as has also been reported for several titanium alloys including Ti-15Mo [22], Ti-10V-4.5Fe-3Al [23], Ti-6Al-2Sn-4Zr-2Mo [10], Ti-6Al-2Zr-2Sn-3Mo-1Cr-2Nb-0.1Si [24] and Ti-3.5Al-5Mo-6V-3Cr-2Sn-0.5Fe [25]. This difference in mechanical properties between the two phases can be attributed to the finer size scale of β in contrast to α, together with the solid solution strengthening effect imparted by the higher content of β-stabilizers (such as Mo and V) in the β phase. Moreover, it has been proposed that the substitution of Ti atoms by smaller V atoms within the α phase lattice creates additional space, which facilitates dislocation glide under reduced frictional stress, thereby resulting in a softer α phase [26]. As a result, the α phase will deform preferentially when the alloy is under plastic deformation. However, the α phase is constrained by the surrounding harder β phase such that dislocations in α grains are piled up and blocked at phase interfaces. This will generate a large amount of GNDs along phase interfaces to accommodate the strain incompatibility between the two phases [27]. As a result, localized internal stresses will be produced and eventually evolve into a long-range HDI stress which in turn interacts with mobile dislocations [13] and makes them difficult to transfer from α to neighboring β until the latter initiates to yield at a larger applied strain. The HDI strengthening and strain hardening have been proved to be important mechanisms contributing to both strength and ductility of heterostructured metals, where the interfaces play an important role [8].

**Fig 5:**
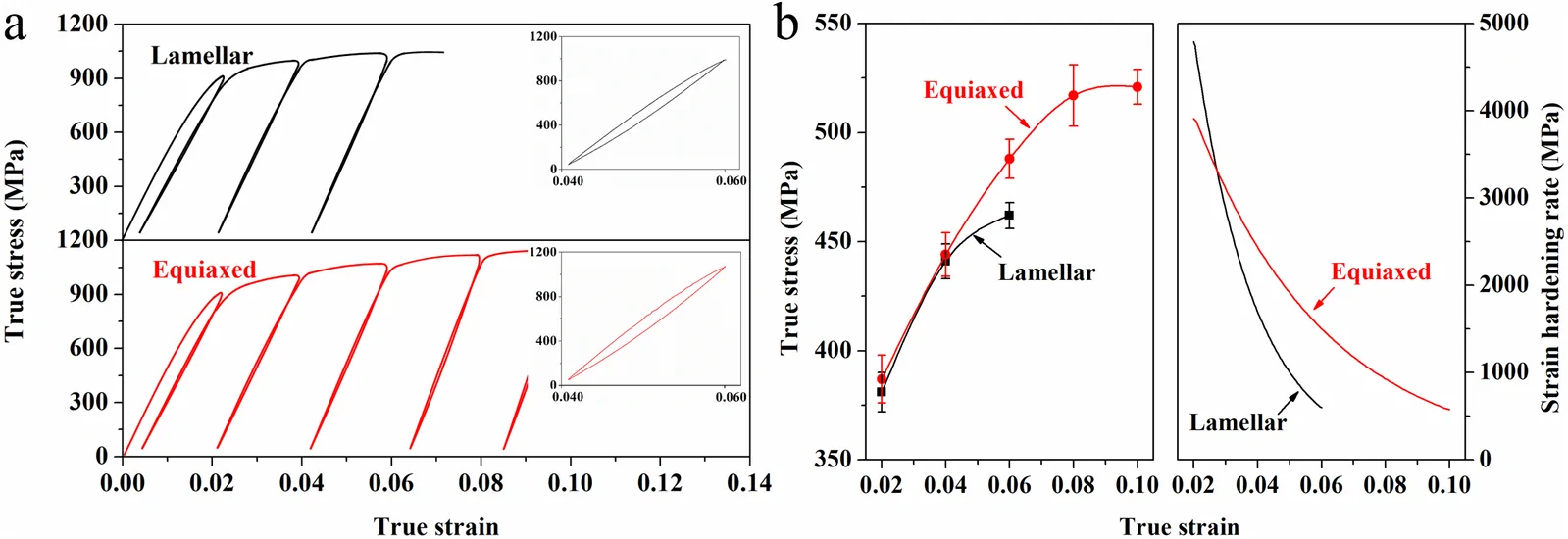
HDI stress analysis for the alloys with lamellar and equiaxed structures. (a) true stress-strain curves of repeated tensile loading and unloading tests; (b) HDI stress and its strain hardening.


The HDI stress originating from structural heterogeneity and inhomogeneous plastic deformation is expected to be affected by the arrangement of phase interface. This led to the study of tensile loading and unloading behaviors on the samples with lamellar and equiaxed structures, from which the influence of microstructural morphology on HDI stress can be quantitatively estimated. Fig 5a shows the true stress-strain curves of the tensile loading and unloading tests. The lamellar-structured sample due to its limited total elongation experienced 3 cycles of unloading and reloading test, while 5 cycles were conducted for the equiaxed sample. Hysteresis loops can be observed in both samples during unloading and reloading cycles, as typically shown in the insets, which indicates a strong Bauschinger effect [19] and therefore the presence of HDI stress. The HDI stress (σ_h_) was calculated by analyzing the hysteresis loop from the following equation [15]:

$$\begin{array}{c} \sigma_{\text{h}}=\frac{\sigma_{\text{u}}+\sigma_{\text{r}}}{\text{2}} \end{array}$$ (1)

where *σ*_u_ and *σ*_r_ are the unloading yield stress and reloading yield stress, respectively. The detailed procedure for the determination of HDI stress was described elsewhere [15]. In the present case, as shown in Fig 5b, the HDI stresses for both samples increase continuously with strain, accounting for more than 40% of maximum flow stress for each unloading-reloading cycle. The HDI stress of the lamellar-structured sample was calculated to be 381 MPa at the tensile strain of 0.02 and it increased up to 462 MPa after tensile strain of 0.06, while for the equiaxed one, the HDI stress was measured to be 387 MPa at the tensile strain of 0.02 and it increased up to 521 MPa after tensile strain of 0.1. Although both samples exhibit almost the same HDI stress magnitude at the early stage of tensile deformation (0.02–0.04), higher HDI stress can be observed in the equiaxed sample as the strain further increases. On the other hand, it is noteworthy that the equiaxed sample shows a higher HDI strain hardening than its lamellar counterpart over a large range of tensile strain. The results indicate that microstructural morphology has an obvious effect on the development of HDI stress, which plays an important role in determining the mechanical properties, especially the ductility in the present case.

It should be pointed out that the two types of microstructures in the present alloy are not strictly heterostructures which require at least 100% strength or hardness differences between the hetero-regions. However, HDI stress is well-developed in this alloy despite the hardness difference between α and β is only about 10%. Similarly, HDI hardening was also reported in a duplex stainless steel [28], where the hardness difference between austenite and ferrite is below 20%. It therefore suggests that the development of HDI stress may be a common phenomenon in dual-phase metals, even if their hardness differences between constituent phases are not so significant.

Conventional view shows that in dual-phase microstructure, the interfaces affecting the back stress mainly display in two aspects: grain boundaries and phase interfaces. In the former aspect, the difference in orientations between grains can lead to strain incompatibilities that produce localized internal stresses against grain boundaries [29]. The grain-to-grain internal stresses developing into a long-range back stress is only pronounced in metals with extremely high density of grain boundaries [30], e.g., in the case of nanostructures [31], while it is not significant in the homogeneous coarse-grained metals. In the latter aspect, however, the back stress comes from the phase-to-phase internal stresses against phase interfaces due to the difference in mechanical properties between the constituent phases, which was frequently reported in dual- or multi-phase metals [2,32] in a large range of grain size scales. It is therefore reasonable to infer that the evolution of HDI stress in the present alloy, which is largely dependent on the development of back stress, is dominated by phase interfaces.

**Fig 6:**
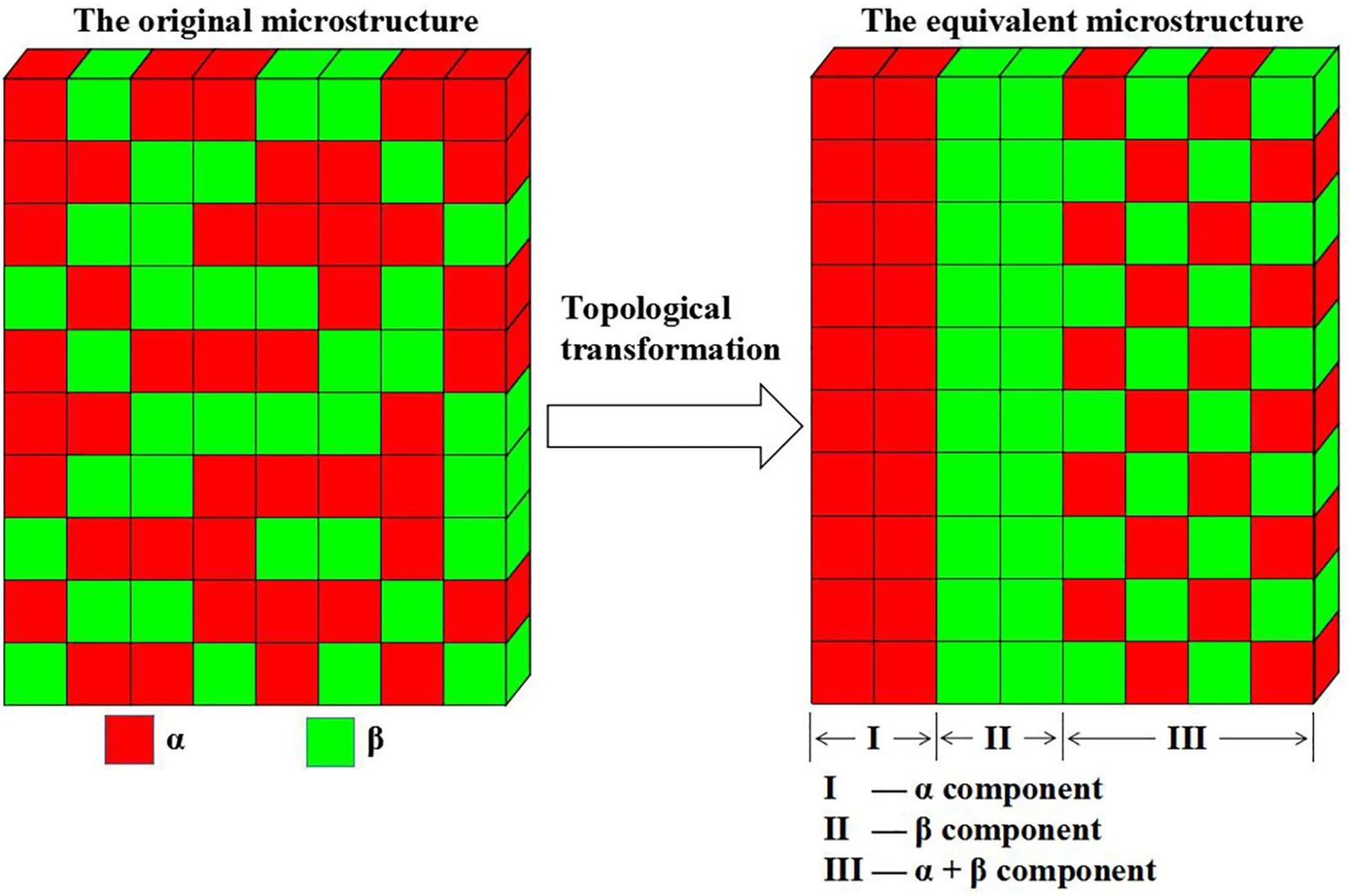
Schematic showing the topological transformation from (left) a giving dual-phase microstructure with the constituent phases randomly distributed to (right) an equivalent microstructure consisting of three well-defined components.


The present study experimentally reveals that the dual-phase microstructure with an equiaxed morphology exhibits stronger HDI strain hardening than that with a lamellar morphology, which is in agreement with a previous finite element methods (FEM) simulation on a heterostructural Cu model consisting of nanostructured and coarse-grained domains [33]. The hetero-interface density has been recognized as the most important parameter difference caused by the change of microstructural morphology, showing a positive correlation with back stress which is proportional to HDI stress [33]. In the present study, the surface-to-volume ratio of phase interface (*s*_V_) was used to characterize the density of phase interface. It was reported that in dual-phase metals *s*_V_ closely relates to the dynamic recovery and therefore shows an influence on the strain hardening ability, in which the higher the value is, the higher the strain hardening rate will be [34]. Based on the topological transformation presented by Fan et al. [35], as shown in Fig 6, a given α + β dual-phase microstructure with any geometrical parameters (e.g., grain size, morphology and phase distribution etc.) can be transformed into an equivalent microstructure which constituted by two single phase (α and β) components including only grains and grain boundaries, and by one dual phase (α + β) component containing phase interfaces alone. Then *s*_V_ in the equivalent microstructure can be expressed as [34]

$$\begin{array}{c} s_{\text{V}}=\frac{\text{3}q_{\alpha \beta }}{{\overset{―}{d}}_{\alpha \beta }} \end{array}$$ (2)

where *q*_αβ_ and ^−^*d*_αβ_ are the volume fraction and the volume-fraction-weighted grain size of the α + β component in the equivalent microstructure, respectively. These two parameters then can be calculated by [35]


$$\begin{array}{c} q_{\alpha \beta }=S_{\alpha }f_{\alpha }\;+\;S_{\beta }f_{\beta } \end{array}$$ (3)

$$\begin{array}{c} {\overset{―}{d}}_{\alpha \beta }=\frac{S_{\alpha }f_{\alpha }d_{\alpha }+S_{\beta }f_{\beta }d_{\beta }}{q_{\alpha \beta }} \end{array}$$ (4)

where *S*_α_ and *S*_β_ are the separations of the α and β phases, respectively. The two parameters of separations can be further expressed as [35]


$$\begin{array}{c} S_{\alpha }=\frac{N_{\text{L}}^{\alpha \beta }}{{\text{2}N}_{\text{L}}^{\alpha \alpha }+N_{\text{L}}^{\alpha \beta }} \end{array}$$ (5)

$$\begin{array}{c} S_{\beta }=\frac{N_{\text{L}}^{\alpha \beta }}{\text{2}N_{\text{L}}^{\beta \beta }+N_{\text{L}}^{\alpha \beta }} \end{array}$$ (6)

where $N_{\text{L}}^{\alpha \alpha }$, $N_{\text{L}}^{\beta \beta }$ and $N_{\text{L}}^{\alpha \beta }$ are the intercept counts of α grain boundaries, β grain boundaries and phase interfaces respectively, with a random line of unit length on a polished plane.

**Fig 7:**
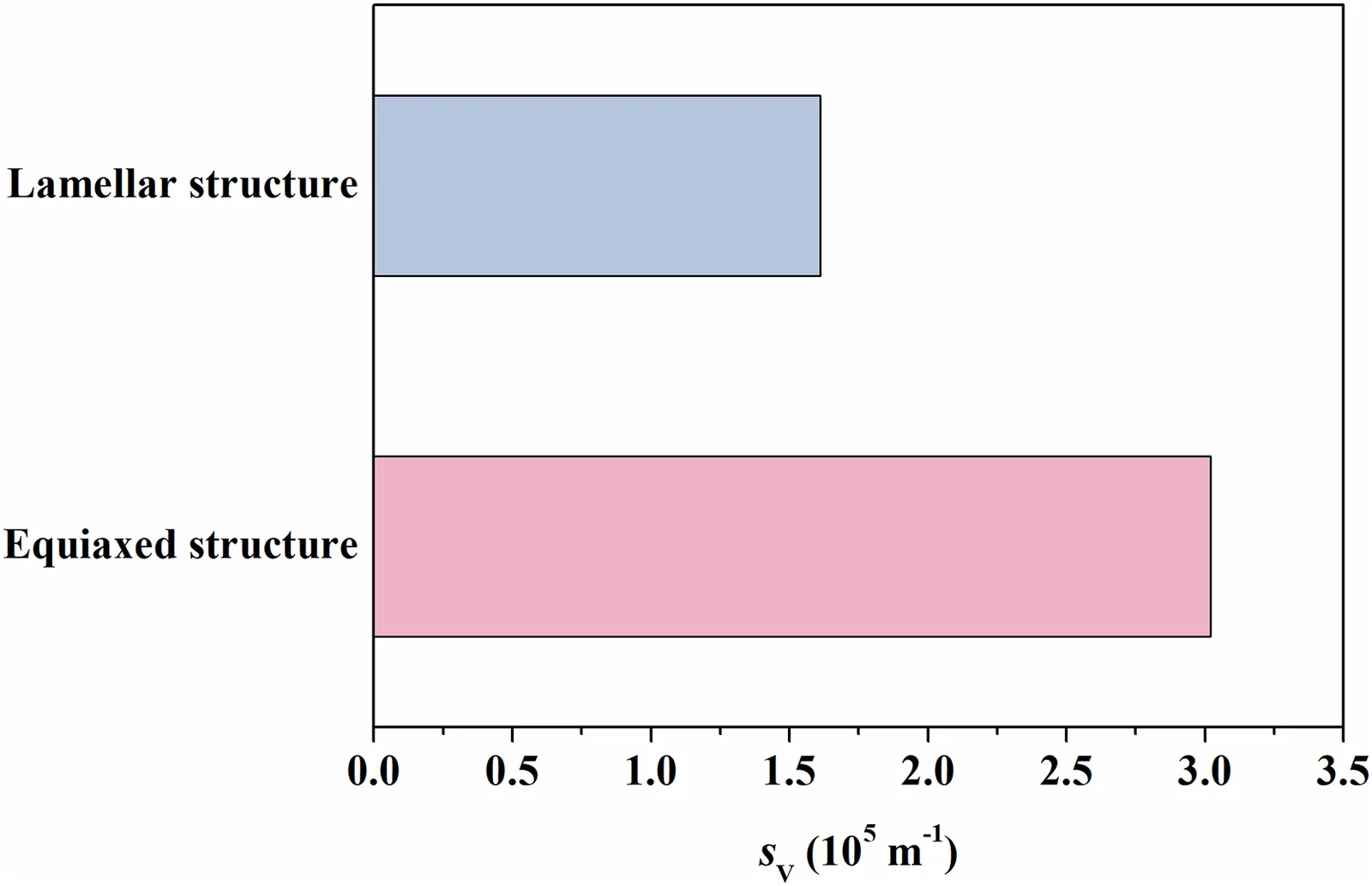
The surface-to-volume ratio of phase interface (*s*_V_) for the alloys with lamellar and equiaxed structures.

By doing the topological transformation and corresponding calculations, as shown in Fig 7, the values of *s*_V_ were estimated to be 1.61 × 10^5^ m^-1^ and 3.02 × 10^5^ m^-1^ for the samples with lamellar and equiaxed structures, respectively. Note that the volume fraction of β in the lamellar-structured sample is only 3.5% lower than that in the equiaxed one, but the latter shows almost 2 times *s*_V_ than the former, indicating the importance of microstructural morphology on the density of phase interface. The deformation heterogeneity of dual-phase microstructure in the phase-scale is mainly manifested as strain gradient concentrations along phase interfaces. Logically it follows that higher density of phase interface can offer more potential sites for GNDs to accumulate and therefore is beneficial to the development of HDI stress.

The effect of microstructural morphology on HDI stress is also expected as different phase interface densities of the initial microstructures may affect the spatial interconnectivity of phase interfaces, which may further affect the strain partitioning between the two phases. It is clear from the microstructural characterization that the equiaxed structure due to its higher density of phase interface shows a more continuous arrangement of phase interface than the lamellar one. That is, the β phase in the equiaxed structure can be considered as a continuous network, where the softer α phases are totally constrained by it. Although deformation usually initiates in α phases, it is difficult for them to change their shape due to the constraint effect by the harder β network. This requires a large amount of GNDs to be generated in the α side near the phase interfaces to accommodate the strain incompatibility between the two phases, which makes it effective to build up HDI stress and then promote the strain hardening arising from the increase of HDI stress.

**Fig 8:**
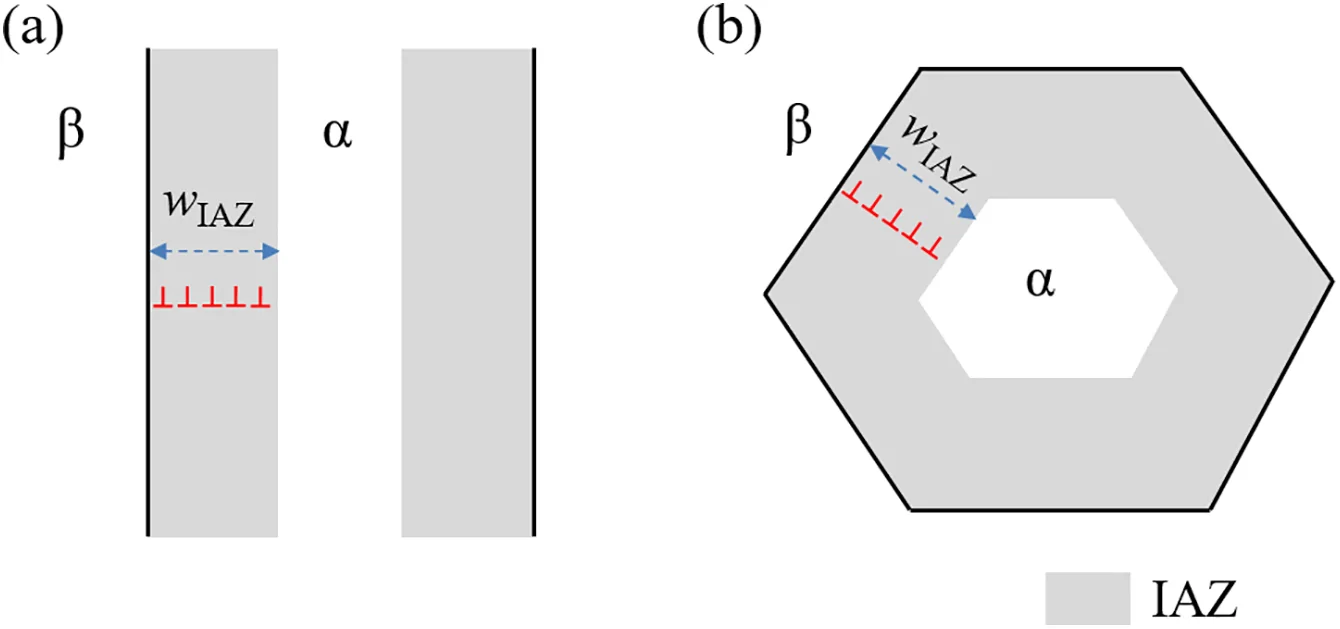
Schematics of IAZs in the softer phases. (a) lamellar structure; (b) equiaxed structure.

Another mechanism for the observed initial microstructure dependent HDI stress evolution can be attributed to the grain size difference enabled by the change of microstructural morphology. Since hetero-deformation leads to a large quantity of GNDs piling-up against the interface, there is an IAZ as a result of the development of GND pileups. The HDI stress is largely affected by IAZ in the softer phase [27], which is schematically illustrated in Fig 8. It suggests that the length scale of IAZ width (*w*_IAZ_) is in the order of a few micrometers [17], depending on the characteristic length of GND pileups. Table 3 lists the typical values of *w*_IAZ_ for several heterostructured metals [16,17,36,37], ranging from ~4 to ~15 μm. It logically follows that the interface spacing of the softer phase plays an important role in allowing the GND pileups to effectively develop and as a result, in the development of HDI stress. Those studies [17,36] also suggested that there is an optimal interface spacing in the softer zone, which is roughly twice of *w*_IAZ_, for achieving the best strain hardening performance. This optimal interface spacing means that the IAZs from neighboring interfaces start, but not yet, to overlap [38]. Whether the interface spacing above or below its optimal value, HDI strain hardening will deteriorate. Such phenomenon can be applied for the present case, despite the optimal interface spacing for the present alloy not being studied. Compared to the α layer thickness in the lamellar structure, the α grain diameter in the equiaxed structure is obviously larger and is also closer to the optimal interface spacings (~ 2*w*_IAZ_) based on the data in Table 3. There has been no report that in heterostructure *w*_IAZ_ is below 4 μm. That is, no evidence that the optimal interface spacing in heterostructure is below ~ 8 μm has been confirmed. When the interface spacing is smaller than its optimal value, HDI strain hardening will decrease with decreasing interface spacing [15]. Back to the present case, the α layer thickness in the lamellar structure is therefore considered being too small to provide sufficient space for GND pileups to fully develop, which limits HDI strain hardening. However, IAZ and its related size effect on HDI stress in the present alloy need to be investigated further.

**Table 3:** Typical values of IAZ width for several heterostructured metals.

Material	*w*_IAZ_ (μm)
Cu/Cu10Zn laminate [17]	5-6
Cu/Cu30Zn laminate [16]	10
Ti/Al composite [36]	15
CoCrNi alloy [37]	4.2

## 4. Conclusions

A near α titanium alloy with lamellar and equiaxed initial microstructures has been used as a model material to study the effect of microstructural morphology on the development of HDI stress during tensile deformation. The main conclusions are summarized as follows:

(i) HDI stresses were confirmed in both lamellar and equiaxed microstructures during tensile tests, which continuously increase with tensile strain. The two types of microstructures show comparable initial HDI stresses but different HDI stress evolutions, where the equiaxed microstructure exhibits higher HDI strain hardening than its lamellar counterpart.
(ii) The difference in the surface-to-volume ratio of phase interface (*s*_V_) between the two microstructures are mainly responsible for their different HDI stress evolutions, where the equiaxed microstructure shows nearly 2 times *s*_V_ than the lamellar one, which can offer more potential sites and better spatial interconnectivity of phase interfaces for GNDs development.
(iii) Microstructural size scale also plays an important role in the development of HDI stress, where the average layer thickness of the lamellar microstructure is considered being too small, as compared to the average grain diameter in the equiaxed one, to provide sufficient space for GND pileups to fully develop, which limits HDI strain hardening.
