# Supplementary material for: Hetero-deformation induced stress and its microstructural morphology dependence in a near-alpha titanium alloy
Source: PLoS One. 2026 Aug 24;21(8):e0356747. doi: 10.1371/journal.pone.0356747 (PMC13502704; doi:10.1371/journal.pone.0356747)
Supplement: Supplementary file 1 [file pone.0356747.s001.docx]

Dear editor,

We appreciate the reviewer’s constructive comments and suggestions on our paper, which are very helpful for us to revise and improve it. We have revised the manuscript according to these comments and suggestions, and all the revisions are highlighted in the manuscript.

In what follows, the response to reviewer’s comments is described in detail.

Reviewer #2 Comments:
1. The authors need to provide a clear justification for selecting a near-α titanium alloy as the model alloy to investigate the influence of microstructural morphology on HDI stress.

**Response:** Two considerations motivated the selection of a near-α titanium alloy as a model material: the tunability of its microstructure and its stability. The near-α titanium alloy is an ideal model system because it allows for the generation of different microstructural morphologies, including both fully lamellar and equiaxed structures, through simple variations in thermomechanical processing without significantly altering the overall chemical composition or volume fraction of the constituent phases. This allows us to isolate the effect of morphology on HDI stress while minimizing confounding variables such as phase fraction or alloy partitioning. The microstructure of this alloy is dominated by the α phase, with a very low content of β phase and almost no β→α' martensitic transformation at room temperature. This characteristic can eliminate the potential influence of phase transformation on HDI stress during deformation, enabling a pure investigation of the influence of microstructural morphology on HDI stress. Furthermore, this selection establishes a critical reference framework for our ongoing studies on titanium alloys with higher compositional complexity. The justification for selecting a near-α titanium alloy as the research subject has been added to the revised manuscript (Lines 85-98 highlighted).

2. In the Materials and Methods section, the authors describe the β phase as having a face-centered cubic crystal structure, which is incorrect. The authors are strongly encouraged to carefully review the relevant literature on titanium alloys and revise the manuscript accordingly to avoid such fundamental inaccuracies.

**Response:** We have carefully checked for similar typos and made corresponding corrections (Line 86 highlighted).

3. The authors should clearly describe the processing route or heat treatment conditions used to obtain the equiaxed and lamellar microstructures. Providing details such as the specific heat-treatment parameters (temperature, holding time, and cooling method) would improve the reproducibility and clarity of the experimental methodology.

**Response:** The preparation details for the two initial microstructures have been added to the Materials and Methods section (Lines 100-101 and 103-106 highlighted).

4. In the Materials and Methods section, authors should specify the operating accelerating voltage used during the SEM and EBSD analyses.

**Response:** The accelerating voltage used for SEM/EBSD observations has been specified in the Materials and Methods section (Lines 110-111 highlighted).

5. The authors should specify the strain rate used during the tensile testing. This is an important experimental parameter that can significantly influence the deformation behavior and mechanical response of the material.

**Response:** The strain rate used for tensile tests has been specified in the Materials and Methods section (Line 120 highlighted).

6. The authors should clarify how the hardness versus strain values were obtained. It is not clear whether the measurements were performed using interrupted tensile testing followed by hardness measurements at different strain levels. A detailed description of the experimental procedure would improve the clarity and reproducibility of the result.

**Response:** The hardness of constituent phases was measured in the central region of the tensile specimen's gauge section during interrupted tensile tests at different strain levels, as described in the revised manuscript (Lines 125-127 highlighted).

7. Figure 1: The individual subfigures should be clearly labeled with appropriate letters (e.g., a, b, c, d) to improve readability and facilitate discussion in the text.

**Response:** The four subfigures in Fig. 1 have been labeled a, b, c and d in the revised manuscript.

8. Figure 1: The description of Figure 1 is not sufficiently clear. The authors should clearly explain the results presented in this figure. It is unclear what type of SEM image (SE/BSE) is shown in Fig. 1(a) and which specific EBSD map is presented. The figure caption and corresponding discussion in the text should be revised to clearly describe each subfigure.

**Response:** Each subfigure in Fig. 1 has been described in the figure caption (Lines 149-151 highlighted) and the corresponding discussion in the text (Lines 131-133 and 136-137 highlighted).

9. The manuscript reports an “average grain diameter of 2 μm” for the β phase with a lamellar/layered morphology, for which the physical meaning of grain diameter is unclear. While the use of grain diameter is appropriate for the equiaxed α phase, the β phase still retains a lamellar morphology. The authors should clarify how this value was determined and consider using more appropriate parameters such as lamellar thickness, aspect ratio or layer spacing to describe the β phase.

**Response:** Following this comment, we re-analyzed the EBSD results. The size of the β phase in the equiaxed initial microstructure was re-measured and expressed as layer thickness. Corresponding revisions have been made accordingly in both Table 1 (highlighted) and the text (Line 141 highlighted).

10. Line 142: The authors state that “continuous flow can be observed in both curves,” but the meaning of “continuous flow” in the context of the stress–strain curves is unclear. The authors should clarify what is meant by this term and explain the specific deformation behavior being referred to.

**Response:** A more appropriate description—"continuous yielding without an obvious yield plateau"—has been adopted in the revised manuscript (Lines 156-157 highlighted).

11. Lines 170–171, 199–201, 330, 332 and Fig. 3: In titanium alloys, the HCP α phase is typically harder than the β phase. However, in this work, the authors report that “β phases always exhibit higher average hardnesses than their α counterparts,” and Fig. 3 also shows higher hardness for the β phase. The authors should provide a clear explanation for this observation and discuss the possible microstructural or compositional factors responsible for the higher hardness of the β phase in this study.

**Response:** The α phase has been reported to be softer than the β phase in some Ti alloys (Mater Charact. 2020; 167: 110521, J Alloys Compd. 2017; 724: 112-120, Mater Sci Eng. 2010; 527: 1480-1488 and so on). Two factors are mainly responsible for this difference in hardness between the two phases in the present alloy. One is the finer size scale of β in contrast to α, the other is the solid solution strengthening effect imparted by the higher content of β-stabilizers (primarily Mo and V) in the β phase. Dareh Baghi et al. (Metals. 2022; 12: 1462) also suggested that apart from the solid solution strengthening effect of V on the β phase, the space created by smaller V atoms substituting Ti atoms in the α phase crystal lattice allows dislocations to move with reduced frictional stresses, leading to a softer α phase. Clear explanation and discussion of this phenomenon have been provided in the revised manuscript (Lines 214–225, highlighted), and the corresponding references [22-26] have also been added to further support it.

12. Figure 4: The authors should clearly specify what type of TEM image is presented in Fig. 4. In addition, the beam direction (zone axis) used during imaging of the dislocations should be indicated, as this information is important for proper interpretation of the dislocation structures.

**Response:** The TEM observation was carried out in bright-field mode with the electron beam approximately parallel to the [11-20] zone axis. This condition has been described in the caption of Fig. 4 in the revised manuscript (Lines 211–212, highlighted).

13. Figure 5: The scale bar of the inset image is not clearly visible. The authors should revise the figure to ensure that the scale is clearly readable.

**Response:** Figure 5 has been revised to make the scale bar of the inset image clearly readable.
