# Supplementary material for: Hetero-deformation induced stress and its microstructural morphology dependence in a near-alpha titanium alloy
Source: PLoS One. 2026 Aug 24;21(8):e0356747. doi: 10.1371/journal.pone.0356747 (PMC13502704; doi:10.1371/journal.pone.0356747)
Supplement: Peer review history

# Peer review: Decision Letter 0

**DOI:** 10.1371/journal.pone.0356747.r001

**Authors:** Arun Devaraj

31 Mar 2026

Dear Dr. Xie,

Thank you for submitting your manuscript to PLOS ONE. After careful consideration, we feel that it has merit but does not fully meet PLOS ONE’s publication criteria as it currently stands. Therefore, we invite you to submit a revised version of the manuscript that addresses the points raised during the review process.

Please submit your revised manuscript by May 15 2026 11:59PM. If you will need more time than this to complete your revisions, please reply to this message or contact the journal office at plosone@plos.org. When you're ready to submit your revision, log on to https://www.editorialmanager.com/pone/ and select the 'Submissions Needing Revision' folder to locate your manuscript file.

- A letter that responds to each point raised by the academic editor and reviewer(s). You should upload this letter as a separate file labeled 'Response to Reviewers'.
- A marked-up copy of your manuscript that highlights changes made to the original version. You should upload this as a separate file labeled 'Revised Manuscript with Track Changes'.
- An unmarked version of your revised paper without tracked changes. You should upload this as a separate file labeled 'Manuscript'.

If you would like to make changes to your financial disclosure, please include your updated statement in your cover letter. Guidelines for resubmitting your figure files are available below the reviewer comments at the end of this letter.

If applicable, we recommend that you deposit your laboratory protocols in protocols.io to enhance the reproducibility of your results. Protocols.io assigns your protocol its own identifier (DOI) so that it can be cited independently in the future. For instructions see: https://journals.plos.org/plosone/s/submission-guidelines#loc-laboratory-protocols. Additionally, PLOS ONE offers an option for publishing peer-reviewed Lab Protocol articles, which describe protocols hosted on protocols.io. Read more information on sharing protocols at https://plos.org/protocols?utm_medium=editorial-email&utm_source=authorletters&utm_campaign=protocols.

As the corresponding author, your ORCID iD is verified in the submission system and will appear in the published article. PLOS supports the use of ORCID, and we encourage all coauthors to register for an ORCID iD and use it as well. Please encourage your coauthors to verify their ORCID iD within the submission system before final acceptance, as unverified ORCID iDs will not appear in the published article. *Only*  the individual author can complete the verification step; PLOS staff *cannot*  verify ORCID iDs on behalf of authors.

We look forward to receiving your revised manuscript.

Kind regards,

Arun Devaraj

Academic Editor

PLOS One

Journal requirements:

When submitting your revision, we need you to address these additional requirements.

1. Please ensure that your manuscript meets PLOS ONE's style requirements, including those for file naming. The PLOS ONE style templates can be found at

https://journals.plos.org/plosone/s/file?id=wjVg/PLOSOne_formatting_sample_main_body.pdf and

https://journals.plos.org/plosone/s/file?id=ba62/PLOSOne_formatting_sample_title_authors_affiliations.pdf

2. Thank you for stating the following financial disclosure:

“This work was supported by the Key Laboratory of the Evaluation and Monitoring of Southwest Land Resources (Ministry of Education, TDSY202409).”

Please state what role the funders took in the study.  If the funders had no role, please state: "The funders had no role in study design, data collection and analysis, decision to publish, or preparation of the manuscript."

If this statement is not correct you must amend it as needed.

Please include this amended Role of Funder statement in your cover letter; we will change the online submission form on your behalf.

3. In the online submission form you indicate that your data is not available for proprietary reasons and have provided a contact point for accessing this data. Please note that your current contact point is a co-author on this manuscript. According to our Data Policy, the contact point must not be an author on the manuscript and must be an institutional contact, ideally not an individual. Please revise your data statement to a non-author institutional point of contact, such as a data access or ethics committee, and send this to us via return email. Please also include contact information for the third party organization, and please include the full citation of where the data can be found.

4. We note that you have indicated that there are restrictions to data sharing for this study. PLOS only allows data to be available upon request if there are legal or ethical restrictions on sharing data publicly. For more information on unacceptable data access restrictions, please see http://journals.plos.org/plosone/s/data-availability#loc-unacceptable-data-access-restrictions.

Before we proceed with your manuscript, please address the following prompts:

a) If there are ethical or legal restrictions on sharing a de-identified data set, please explain them in detail (e.g., data contain potentially identifying or sensitive patient information, data are owned by a third-party organization, etc.) and who has imposed them (e.g., a Research Ethics Committee or Institutional Review Board, etc.). Please also provide contact information for a data access committee, ethics committee, or other institutional body to which data requests may be sent.

b) If there are no restrictions, please upload the minimal anonymized data set necessary to replicate your study findings to a stable, public repository and provide us with the relevant URLs, DOIs, or accession numbers. For a list of recommended repositories, please see

https://journals.plos.org/plosone/s/recommended-repositories. You also have the option of uploading the data as Supporting Information files, but we would recommend depositing data directly to a data repository if possible.

We will update your Data Availability statement on your behalf to reflect the information you provide.

5. PLOS requires an ORCID iD for the corresponding author in Editorial Manager on papers submitted after December 6th, 2016. Please ensure that you have an ORCID iD and that it is validated in Editorial Manager. To do this, go to ‘Update my Information’ (in the upper left-hand corner of the main menu), and click on the Fetch/Validate link next to the ORCID field. This will take you to the ORCID site and allow you to create a new iD or authenticate a pre-existing iD in Editorial Manager.

If the reviewer comments include a recommendation to cite specific previously published works, please review and evaluate these publications to determine whether they are relevant and should be cited. There is no requirement to cite these works unless the editor has indicated otherwise.

[Note: HTML markup is below. Please do not edit.]

Reviewers' comments:

Reviewer's Responses to Questions

**Comments to the Author**

1. Is the manuscript technically sound, and do the data support the conclusions?

Reviewer #1: Yes

Reviewer #2: Partly

**********

2. Has the statistical analysis been performed appropriately and rigorously?

Reviewer #1: Yes

Reviewer #2: I Don't Know

**********

3. Have the authors made all data underlying the findings in their manuscript fully available?

The PLOS Data policy

Reviewer #1: Yes

Reviewer #2: Yes

**********

4. Is the manuscript presented in an intelligible fashion and written in standard English?

Reviewer #1: Yes

Reviewer #2: Yes

**********

Reviewer #1: The manuscript describe a technically sound piece of scientific research with data that supports the conclusions. Experiments have been conducted rigorously. The conclusions drawn appropriately based on the data presented.

Reviewer #2: In this manuscript, the authors investigated the development of hetero-deformation induced (HDI) stress and the influence of microstructural morphology in a near-α titanium alloy with two distinct initial microstructures: lamellar and equiaxed. They reported relatively enhanced HDI stress in equiaxed microstructure compared to its lamellar one, which was attributed to the higher surface-to-volume ratio of phase interface and higher size scale of the equiaxed microstructure. This study is important to correlate the interrelationship between morphology and HDI stress. However, the manuscript is not recommended for publication in its current form. The authors need to address the following comments.

1. The authors need to provide a clear justification for selecting a near-α titanium alloy as the model alloy to investigate the influence of microstructural morphology on HDI stress.

2. In the Materials and Methods section, the authors describe the β phase as having a face-centered cubic crystal structure, which is incorrect. The authors are strongly encouraged to carefully review the relevant literature on titanium alloys and revise the manuscript accordingly to avoid such fundamental inaccuracies.

3. The authors should clearly describe the processing route or heat treatment conditions used to obtain the equiaxed and lamellar microstructures. Providing details such as the specific heat-treatment parameters (temperature, holding time, and cooling method) would improve the reproducibility and clarity of the experimental methodology.

4. In the Materials and Methods section, authors should specify the operating accelerating voltage used during the SEM and EBSD analyses.

5. The authors should specify the strain rate used during the tensile testing. This is an important experimental parameter that can significantly influence the deformation behavior and mechanical response of the material.

6. The authors should clarify how the hardness versus strain values were obtained. It is not clear whether the measurements were performed using interrupted tensile testing followed by hardness measurements at different strain levels. A detailed description of the experimental procedure would improve the clarity and reproducibility of the result.

7. Figure 1: The individual subfigures should be clearly labeled with appropriate letters (e.g., a, b, c, d) to improve readability and facilitate discussion in the text.

8. Figure 1: The description of Figure 1 is not sufficiently clear. The authors should clearly explain the results presented in this figure. It is unclear what type of SEM image (SE/BSE) is shown in Fig. 1(a) and which specific EBSD map is presented. The figure caption and corresponding discussion in the text should be revised to clearly describe each subfigure.

9. The manuscript reports an “average grain diameter of 2 μm” for the β phase with a lamellar/layered morphology, for which the physical meaning of grain diameter is unclear. While the use of grain diameter is appropriate for the equiaxed α phase, the β phase still retains a lamellar morphology. The authors should clarify how this value was determined and consider using more appropriate parameters such as lamellar thickness, aspect ratio or layer spacing to describe the β phase.

10. Line 142: The authors state that “continuous flow can be observed in both curves,” but the meaning of “continuous flow” in the context of the stress–strain curves is unclear. The authors should clarify what is meant by this term and explain the specific deformation behavior being referred to.

11. Lines 170–171, 199–201, 330, 332 and Fig. 3: In titanium alloys, the HCP α phase is typically harder than the β phase. However, in this work, the authors report that “β phases always exhibit higher average hardnesses than their α counterparts,” and Fig. 3 also shows higher hardness for the β phase. The authors should provide a clear explanation for this observation and discuss the possible microstructural or compositional factors responsible for the higher hardness of the β phase in this study.

12. Figure 4: The authors should clearly specify what type of TEM image is presented in Fig. 4. In addition, the beam direction (zone axis) used during imaging of the dislocations should be indicated, as this information is important for proper interpretation of the dislocation structures.

13. Figure 5: The scale bar of the inset image is not clearly visible. The authors should revise the figure to ensure that the scale is clearly readable.

**********

what does this mean?). If published, this will include your full peer review and any attached files.

If you choose “no”, your identity will remain anonymous but your review may still be made public.

**Do you want your identity to be public for this peer review?**  For information about this choice, including consent withdrawal, please see our Privacy Policy

Reviewer #1: **Yes:**  Arunangsu Das, Ph.D.

Reviewer #2: No

**********

[NOTE: If reviewer comments were submitted as an attachment file, they will be attached to this email and accessible via the submission site. Please log into your account, locate the manuscript record, and check for the action link "View Attachments". If this link does not appear, there are no attachment files.]

To ensure your figures meet our technical requirements, please review our figure guidelines: https://journals.plos.org/plosone/s/figures

You may also use PLOS’s free figure tool, NAAS, to help you prepare publication quality figures: https://journals.plos.org/plosone/s/figures#loc-tools-for-figure-preparation.

NAAS will assess whether your figures meet our technical requirements by comparing each figure against our figure specifications.

# Author comment: Author response to Decision Letter 1

**DOI:** 10.1371/journal.pone.0356747.r002

27 Apr 2026

Reviewer #2 Comments:

1. The authors need to provide a clear justification for selecting a near-α titanium alloy as the model alloy to investigate the influence of microstructural morphology on HDI stress.

Response: Two considerations motivated the selection of a near-α titanium alloy as a model material: the tunability of its microstructure and its stability. The near-α titanium alloy is an ideal model system because it allows for the generation of different microstructural morphologies, including both fully lamellar and equiaxed structures, through simple variations in thermomechanical processing without significantly altering the overall chemical composition or volume fraction of the constituent phases. This allows us to isolate the effect of morphology on HDI stress while minimizing confounding variables such as phase fraction or alloy partitioning. The microstructure of this alloy is dominated by the α phase, with a very low content of β phase and almost no β→α' martensitic transformation at room temperature. This characteristic can eliminate the potential influence of phase transformation on HDI stress during deformation, enabling a pure investigation of the influence of microstructural morphology on HDI stress. Furthermore, this selection establishes a critical reference framework for our ongoing studies on titanium alloys with higher compositional complexity. The justification for selecting a near-α titanium alloy as the research subject has been added to the revised manuscript (Lines 85-98 highlighted).

2. In the Materials and Methods section, the authors describe the β phase as having a face-centered cubic crystal structure, which is incorrect. The authors are strongly encouraged to carefully review the relevant literature on titanium alloys and revise the manuscript accordingly to avoid such fundamental inaccuracies.

Response: We have carefully checked for similar typos and made corresponding corrections (Line 86 highlighted).

3. The authors should clearly describe the processing route or heat treatment conditions used to obtain the equiaxed and lamellar microstructures. Providing details such as the specific heat-treatment parameters (temperature, holding time, and cooling method) would improve the reproducibility and clarity of the experimental methodology.

Response: The preparation details for the two initial microstructures have been added to the Materials and Methods section (Lines 100-101 and 103-106 highlighted).

4. In the Materials and Methods section, authors should specify the operating accelerating voltage used during the SEM and EBSD analyses.

Response: The accelerating voltage used for SEM/EBSD observations has been specified in the Materials and Methods section (Lines 110-111 highlighted).

5. The authors should specify the strain rate used during the tensile testing. This is an important experimental parameter that can significantly influence the deformation behavior and mechanical response of the material.

Response: The strain rate used for tensile tests has been specified in the Materials and Methods section (Line 120 highlighted).

6. The authors should clarify how the hardness versus strain values were obtained. It is not clear whether the measurements were performed using interrupted tensile testing followed by hardness measurements at different strain levels. A detailed description of the experimental procedure would improve the clarity and reproducibility of the result.

Response: The hardness of constituent phases was measured in the central region of the tensile specimen's gauge section during interrupted tensile tests at different strain levels, as described in the revised manuscript (Lines 125-127 highlighted).

7. Figure 1: The individual subfigures should be clearly labeled with appropriate letters (e.g., a, b, c, d) to improve readability and facilitate discussion in the text.

Response: The four subfigures in Fig. 1 have been labeled a, b, c and d in the revised manuscript.

8. Figure 1: The description of Figure 1 is not sufficiently clear. The authors should clearly explain the results presented in this figure. It is unclear what type of SEM image (SE/BSE) is shown in Fig. 1(a) and which specific EBSD map is presented. The figure caption and corresponding discussion in the text should be revised to clearly describe each subfigure.

Response: Each subfigure in Fig. 1 has been described in the figure caption (Lines 149-151 highlighted) and the corresponding discussion in the text (Lines 131-133 and 136-137 highlighted).

9. The manuscript reports an “average grain diameter of 2 μm” for the β phase with a lamellar/layered morphology, for which the physical meaning of grain diameter is unclear. While the use of grain diameter is appropriate for the equiaxed α phase, the β phase still retains a lamellar morphology. The authors should clarify how this value was determined and consider using more appropriate parameters such as lamellar thickness, aspect ratio or layer spacing to describe the β phase.

Response: Following this comment, we re-analyzed the EBSD results. The size of the β phase in the equiaxed initial microstructure was re-measured and expressed as layer thickness. Corresponding revisions have been made accordingly in both Table 1 (highlighted) and the text (Line 141 highlighted).

10. Line 142: The authors state that “continuous flow can be observed in both curves,” but the meaning of “continuous flow” in the context of the stress–strain curves is unclear. The authors should clarify what is meant by this term and explain the specific deformation behavior being referred to.

Response: A more appropriate description—"continuous yielding without an obvious yield plateau"—has been adopted in the revised manuscript (Lines 156-157 highlighted).

11. Lines 170–171, 199–201, 330, 332 and Fig. 3: In titanium alloys, the HCP α phase is typically harder than the β phase. However, in this work, the authors report that “β phases always exhibit higher average hardnesses than their α counterparts,” and Fig. 3 also shows higher hardness for the β phase. The authors should provide a clear explanation for this observation and discuss the possible microstructural or compositional factors responsible for the higher hardness of the β phase in this study.

Response: The α phase has been reported to be softer than the β phase in some Ti alloys (Mater Charact. 2020; 167: 110521, J Alloys Compd. 2017; 724: 112-120, Mater Sci Eng. 2010; 527: 1480-1488 and so on). Two factors are mainly responsible for this difference in hardness between the two phases in the present alloy. One is the finer size scale of β in contrast to α, the other is the solid solution strengthening effect imparted by the higher content of β-stabilizers (primarily Mo and V) in the β phase. Dareh Baghi et al. (Metals. 2022; 12: 1462) also suggested that apart from the solid solution strengthening effect of V on the β phase, the space created by smaller V atoms substituting Ti atoms in the α phase crystal lattice allows dislocations to move with reduced frictional stresses, leading to a softer α phase. Clear explanation and discussion of this phenomenon have been provided in the revised manuscript (Lines 214–225, highlighted), and the corresponding references [22-26] have also been added to further support it.

12. Figure 4: The authors should clearly specify what type of TEM image is presented in Fig. 4. In addition, the beam direction (zone axis) used during imaging of the dislocations should be indicated, as this information is important for proper interpretation of the dislocation structures.

Response: The TEM observation was carried out in bright-field mode with the electron beam approximately parallel to the [11-20] zone axis. This condition has been described in the caption of Fig. 4 in the revised manuscript (Lines 211–212, highlighted).

13. Figure 5: The scale bar of the inset image is not clearly visible. The authors should revise the figure to ensure that the scale is clearly readable.

Response: Figure 5 has been revised to make the scale bar of the inset image clearly readable.

Attachment Submitted filename: Response to Reviewers.docx

# Peer review: Decision Letter 1

**DOI:** 10.1371/journal.pone.0356747.r003

**Authors:** Sridhar Babu B

7 Aug 2026

Hetero-deformation induced stress and its microstructural morphology dependence in a near-alpha titanium alloy

PONE-D-25-53181R1

Dear Dr. Xie,

We’re pleased to inform you that your manuscript has been judged scientifically suitable for publication and will be formally accepted for publication once it meets all outstanding technical requirements.

Within one week, you’ll receive an e-mail detailing the required amendments. When these have been addressed, you’ll receive a formal acceptance letter and your manuscript will be scheduled for publication.

An invoice will be generated when your article is formally accepted. Please note, if your institution has a publishing partnership with PLOS and your article meets the relevant criteria, all or part of your publication costs will be covered. Please make sure your user information is up-to-date by logging into Editorial Manager at Editorial Manager® and clicking the ‘Update My Information' link at the top of the page. For questions related to billing, please contact billing support.

If your institution or institutions have a press office, please notify them about your upcoming paper to help maximize its impact. If they’ll be preparing press materials, please inform our press team as soon as possible -- no later than 48 hours after receiving the formal acceptance. Your manuscript will remain under strict press embargo until 2 pm Eastern Time on the date of publication. For more information, please contact onepress@plos.org.

Kind regards,

Sridhar Babu B

Academic Editor

PLOS One

Additional Editor Comments (optional):

Reviewers' comments:

Reviewer's Responses to Questions

**Comments to the Author**

Reviewer #1: All comments have been addressed

Reviewer #2: All comments have been addressed

**********

2. Is the manuscript technically sound, and do the data support the conclusions?

Reviewer #1: Yes

Reviewer #2: Yes

**********

3. Has the statistical analysis been performed appropriately and rigorously?

Reviewer #1: Yes

Reviewer #2: Yes

**********

4. Have the authors made all data underlying the findings in their manuscript fully available?

The PLOS Data policy

Reviewer #1: Yes

Reviewer #2: Yes

**********

5. Is the manuscript presented in an intelligible fashion and written in standard English?

Reviewer #1: Yes

Reviewer #2: Yes

**********

Reviewer #1: The manuscript describe a technically sound piece of scientific research with data that supports the conclusions. Experiments have been conducted rigorously. The conclusions are drawn appropriately based on the data presented.

Reviewer #2: (No Response)

**********

what does this mean?). If published, this will include your full peer review and any attached files.

If you choose “no”, your identity will remain anonymous but your review may still be made public.

**Do you want your identity to be public for this peer review?**  For information about this choice, including consent withdrawal, please see our Privacy Policy

Reviewer #1: **Yes:**  Arunangsu Das, Ph.D.

Reviewer #2: No

**********

# Editor report: Acceptance letter

**DOI:** 10.1371/journal.pone.0356747.r004

**Authors:** Sridhar Babu B

PONE-D-25-53181R1

PLOS One

Dear Dr. Xie,

I'm pleased to inform you that your manuscript has been deemed suitable for publication in PLOS One. Congratulations! Your manuscript is now being handed over to our production team.

At this stage, our production department will prepare your paper for publication. This includes ensuring the following:

* All references, tables, and figures are properly cited

* All relevant supporting information is included in the manuscript submission,

* There are no issues that prevent the paper from being properly typeset

You will receive further instructions from the production team, including instructions on how to review your proof when it is ready. Please keep in mind that we are working through a large volume of accepted articles, so please give us a few days to review your paper and let you know the next and final steps.

Lastly, if your institution or institutions have a press office, please let them know about your upcoming paper now to help maximize its impact. If they'll be preparing press materials, please inform our press team within the next 48 hours. Your manuscript will remain under strict press embargo until 2 pm Eastern Time on the date of publication. For more information, please contact onepress@plos.org.

You will receive an invoice from PLOS for your publication fee after your manuscript has reached the completed accept phase. If you receive an email requesting payment before acceptance or for any other service, this may be a phishing scheme. Learn how to identify phishing emails and protect your accounts at https://explore.plos.org/phishing.

If we can help with anything else, please email us at customercare@plos.org.

Thank you for submitting your work to PLOS One and supporting open access.

Kind regards,

PLOS ONE Editorial Office Staff

on behalf of

Dr. Sridhar Babu B

Academic Editor

PLOS One
